# Perceived Message Effectiveness of the Meatless Monday Campaign: An Experiment with US Adults

**DOI:** 10.2105/AJPH.2022.306766

**Published:** 2022-03-24

**Authors:** Hannah Rayala, Natalia Rebolledo, Marissa G. Hall, Lindsey Smith Taillie

**Affiliations:** 1 Department of Nutrition, Gillings School of Global Public Health, University of North Carolina at Chapel Hill, Chapel Hill, NC, USA; 2 Department of Health Behavior, Gillings School of Global Public Health, University of North Carolina at Chapel Hill, Chapel Hill, NC, USA; 3 Carolina Population Center, University of North Carolina at Chapel Hill, Chapel Hill, NC, USA; 4 Lineberger Comprehensive Cancer Center, University of North Carolina at Chapel Hill, Chapel Hill, NC, USA

## Abstract

Given the negative health and environmental impacts of red meat consumption, reducing red meat intake in the U.S. is important for both human and planetary well-being. To experimentally evaluate the impact of health-focused and environment-focused messages from the Meatless Monday campaign, we conducted an online randomized experiment among U.S. adults aged 18 or older (n=1,244). Compared to control messages, health-focused and environment-focused Meatless Monday messages led to significantly higher perceived message effectiveness and increased intention to reduce meat consumption.

Excess consumption of red and processed meat is a growing problem in the United States, where the amount of meat consumed is more than three times the global average.^1^ Given the association between excess meat intake and negative health and environmental outcomes, decreasing meat consumption in high-income countries such as the U.S. is important to reduce the global burden of chronic disease and the negative environmental consequences of meat production.^2^ Mass media campaigns are a promising but untested population-level strategy for reducing meat intake.

## Intervention

In 2003, the Johns Hopkins Bloomberg School of Public Health launched the Meatless Monday campaign with the goal of reducing meat consumption by 15% to promote human and planetary health.^3^ A nationally representative sample of U.S. adults from 2019 found that 42% of respondents were aware of the Meatless Monday campaign, and 21% had participated in Meatless Monday at some point.^4^ The campaign strategy tested in our study consisted of graphics communicating the negative health and environmental impacts of meat consumption. The specific images used were selected based on a combination of image popularity measured by social media shares, and diversity of stimuli in terms of different health and environmental outcomes depicted in the messages and design styles represented (Supplemental Figure 1).

## Purpose

Although Meatless Monday is widely recognized, the campaign has not yet been evaluated for Perceived Message Effectiveness (PME). This measure predicts behavioral change and is often used to vet campaign messages.^5^ In addition, it is unclear whether Meatless Monday campaign messages attract attention, or lead to negative affect, cognitive elaboration, increased social interactions, and intention to reduce red meat intake. All of these constructs are on the pathway from message exposure to behavioral change according to the UNC Warnings Impact Model, which has been used to evaluate other health outcomes (e.g. sugar-sweetened beverage consumption and tobacco use).^6,7^ Furthermore, it is unclear whether consumers’ reactions to Meatless Monday messages vary by their frequency of red meat consumption.

To address these knowledge gaps, our study sought to experimentally evaluate the impact of health- focused and environment-focused messages from the Meatless Monday campaign using constructs predictive of behavioral change through a one-time online survey in a sample of U.S. adults. Additionally, we aimed to understand whether the frequency of red meat consumption moderated the impact of Meatless Monday campaign messages on consumers.

## Implementation (Person, Place, and Time)

Our randomized experiment consisted of a one-time online survey launched from September 2020 to October 2020 through CloudResearch's Prime Panels. The study population consisted of 1,244 U.S. adults aged 18 years or older who could read, write, and speak English and had consumed red meat at least once per week in the past 30 days (Supplemental Table 1). In the overall sample, the mean age was 45 years (SE=0.48) and 27.6% of participants had an annual household income of less than $25,000. Most participants self-identified as White (77.9%) and non-Hispanic (89.0%). The largest proportion of participants were male (52.2%), had obtained at least a college degree (49.1%), and self-identified as Democrats (40.3%).

After eligibility screening and providing electronic informed consent, participants proceeded to the experiment survey, which used a between-subjects’ design. Participants were randomly assigned to one of three trial arms: 1) control messages (which pertained to credit scores), 2) health-focused Meatless Monday messages, or 3) environment-focused Meatless Monday messages and viewed four graphics specific to the trial arm displayed in random order (Supplemental Figure 1). Participants then answered a series of questions about the messages they viewed regarding health concern, environmental concern, discouragement, and unpleasantness which taken together, constituted our primary outcome measure of PME (Supplemental Table 2). Participants were also asked questions regarding attention, negative affect, cognitive elaboration, social interactions, and intention to reduce meat consumption which were all secondary outcome measures in this study (Supplemental Table 3).

## Evaluation

We found that compared to control messages, both health-focused and environment-focused Meatless Monday campaign messages effectively increased PME (Table 1). Additionally, both health-focused and environment-focused messages scored significantly higher in all secondary outcome measures including attention, negative affect, cognitive elaboration, social interactions, and intention to reduce meat consumption. Furthermore, there were no significant differences between health-focused and environmental-focused messages for any of the outcomes. These findings show that relative to control messages, Meatless Monday messages attracted participants’ attention more, increased their negative perception of meat consumption, led them to think about the health and environmental harms of consuming meat, and made participants more interested in talking about the Meatless Monday campaign in their social interactions. Given that these constructs are predictive of behavioral change, these results suggest that widespread communication campaigns such as Meatless Monday are a promising public health strategy to mitigate the negative health and environmental effects of meat consumption. However, further research would benefit from testing these messages on behavioral outcomes, such as purchases and consumption of red and processed meat. Additionally, given that our sample was predominantly white, future studies should examine whether these findings hold in more diverse samples with respect to race and ethnicity.

Following our analysis of meat consumption frequency as a potential moderator of the effect of Meatless Monday messages on PME, we found that among high-frequency meat consumers (i.e. participants who reported consuming red meat once a day or more), neither the health-focused nor the environment-focused messages elicited significantly higher PME compared to the control messages (Table 2). These results appear to be driven by higher ratings of the control messages among frequent meat consumers. It is unclear what drove the higher rating of the control messages within this group, but further investigations on attitudes and values surrounding meat consumption would be valuable in providing insight into effective message designs tailored to reach high-frequency meat consumers.

## Sustainability

By focusing only on eliminating meat one day per week, Meatless Monday provides a more feasible way to reduce meat consumption among current meat consumers, compared to complete elimination diets as seen with vegetarianism and veganism. Although real-world evidence of the impact of the Meatless Monday campaign is nascent, many popular fast-food chains (including McDonald’s, Subway, and Burger King) already offer plant-based options on their menu, and Starbucks has even launched a campaign to provide customers discounts for meatless options on Monday.^8,9^

## Public Health Significance

Our results suggest that the Meatless Monday campaign's health and environmental messages are effective in increasing intention to reduce meat consumption among consumers who are exposed to them. Because previous evidence from behavioral studies has shown that intention to change is one of the strongest predictors of actual behavioral change, national distribution and promotion of the Meatless Monday campaign could have meaningful effects on meat consumption in the U.S.^6,10,11^ While this study shows promise with regards to the perceived effectiveness of the messages, it is important to acknowledge that campaign messages can only be effective if they are aired at sufficient weight to be noticed by the majority of the population over a sustained period.^12^ Overall, our results suggest that widespread implementation of similar initiatives among other popular food chains and through public policy could prove to be a promising and attainable step forward in reducing meat consumption in the U.S. as a solution to mitigate the negative health and environmental impacts of meat production.

## Supplementary Material

Supplemental Material

## Figures and Tables

**Table 1:** Mean PME and secondary outcomes by exposure to Control, Health-focused, and Environment-focused Meatless Monday Messages.

	Control		Health-focused			Environment-focused			
	Mean	SE	Mean	SE	P-value^a^	Mean	SE	P-value^a^	P-value^b^
PME	1.7	0.06	2.8	0.06	<0.001	2.9	0.06	<0.001	1.000
Attention	2.9	0.06	3.3	0.06	<0.001	3.3	0.06	<0.001	1.000
Negative Affect	2.0	0.06	2.5	0.06	<0.001	2.7	0.06	<0.001	0.560
Cognitive Elaboration (Health)	1.7	0.06	3.0	0.07	<0.001	2.9	0.06	<0.001	0.122
Cognitive Elaboration (Environment)	1.8	0.06	2.7	0.07	<0.001	3.1	0.06	<0.001	<0.001
Social Interactions	2.0	0.07	2.6	0.07	<0.001	2.6	0.07	<0.001	1.000
Intention to reduce Meat Consumption	2.3	0.07	2.9	0.07	<0.001	3.0	0.07	<0.001	1.000

SE = Standard Error. PME = Perceived Message Effectiveness. P-values were obtained using Bonferroni correction for 3 comparisons (statistical significance was defined as p<0.016).

a P-value is for the contrast between each Meatless Monday arm messages and the control.

b P-value is for the contrast between the environment-focused arm compared to the heath-focused arm.

**Table 2:** Mean PME by Meat Consumption Frequency for Control, Health-focused messages, and Environment-focused messages groups.

Meat Consumption Frequency	Control		Health-focused			Environment-focused		
	Mean	SE	Mean	SE	P-Value*	Mean	SE	P-Value*
Low meat consumption frequency	1.8	0.10	3.0	0.11	<0.001	3.0	0.11	<0.001
Moderate meat consumption frequency	1.5	0.07	2.8	0.07	<0.001	2.8	0.07	<0.001
High meat consumption frequency	2.4	0.17	2.9	0.15	0.038	2.9	0.14	0.332

Meat consumption frequency was recategorized into a 3 level category for statistical analysis: “low meat consumption” = 1 time a week or less, “moderate meat consumption” = more than 1 time per week but less than 1 time per day, and “high meat consumption” = 1 time a day or more. SE = Standard Error. Means obtained by combining all 4 PME categories using linear regression models. P-values were obtained using Bonferroni correction for 6 comparisons (statistical significance was defined as p<0.008).

* P-value is for the contrast between each Meatless Monday arm messages and the control within level of meat consumption.

Note: p-value for Wald test for interaction of Arm and frequency of meat consumption < 0.001.
