# Supplementary material for: Perceived Message Effectiveness of the Meatless Monday Campaign: An Experiment with US Adults
Source: Am J Public Health. Author manuscript; Available in PMC 2022 May 1. (PMC9010929; doi:10.2105/AJPH.2022.306766)
Supplement: Supplemental Material [file EMS141052-supplement-Supplemental_Material.pdf]

Supplemental Figure 1.

1a. Control condition messages

Always check your credit score.

Remember to check your credit score.

Don't forget to check your credit score.

Be sure to check your credit score.

1b. Health-focused messages

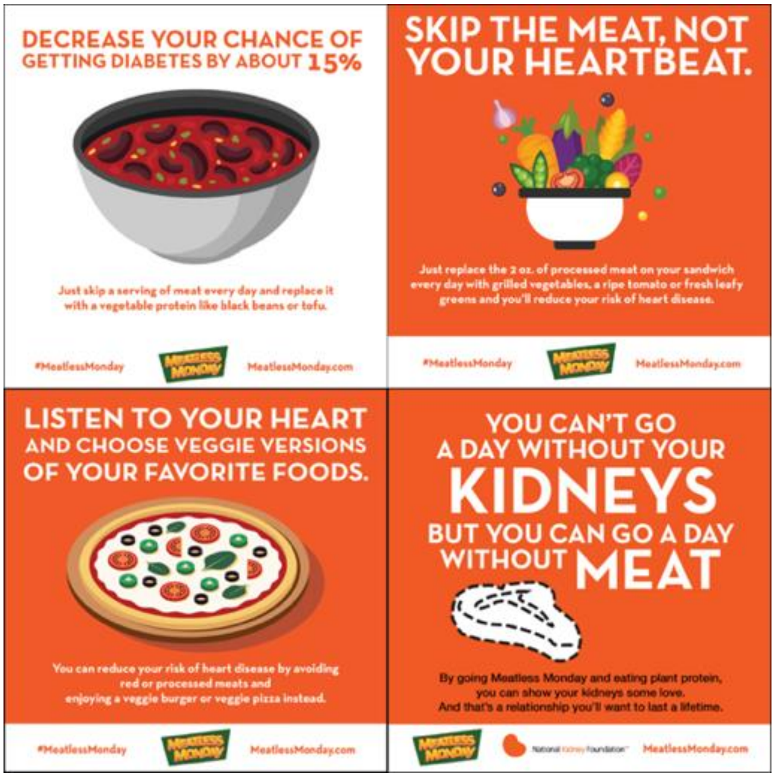

1c. Environment-focused messages

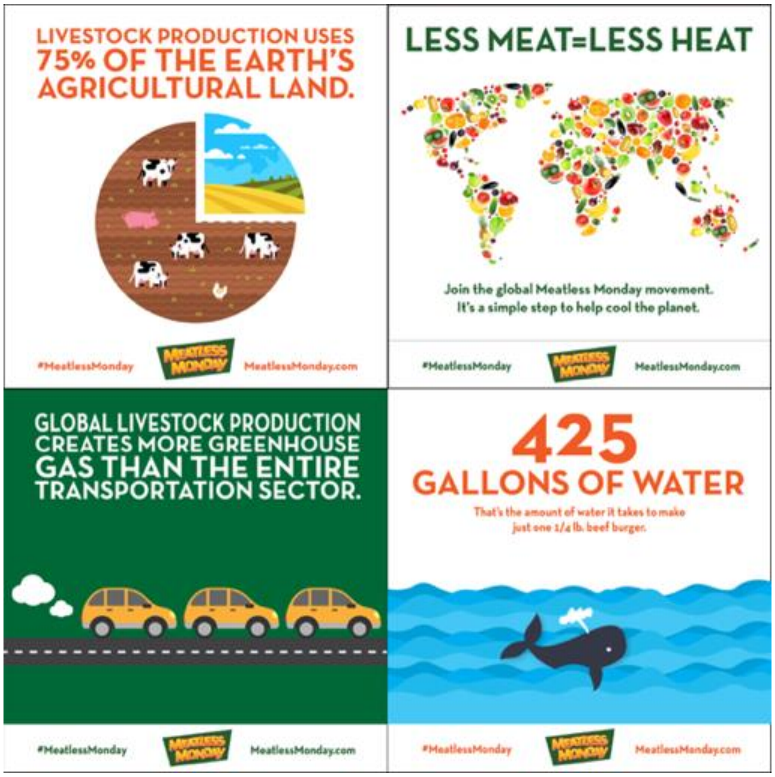

Supplemental Table 1. Participant Characteristics by exposure to Control, Health-focused, and Environment-focused Meatless Monday Messages (n = 1,244).

Characteristics	Overall	Control	Health	Environment	P-Value*
n (%)	1,244	414 (33.3%)	412 (33.1%)	418 (33.6%)	
Age, years					0.755
18–20 years	4.5%	5.1%	5.1%	3.4%	
21–29 years	18.9%	18.6%	17.5%	20.6%	
30–39 years	19.3%	21.8%	18.4%	17.7%	
40–49 years	16.3%	16.5%	15.8%	16.8%	
50–59 years	16.1%	15.5%	16.7%	16.1%	
60+ years	24.8%	22.5%	26.5%	25.4%	
Mean (SE)	44.9 (0.48)	43.8 (0.81)	45.8 (0.84)	45.3 (0.82)	
Gender					0.719
Male	52.2%	51.5%	51.2%	53.8%	
Female	47.0%	48.1%	47.6%	45.4%	
Other	0.8%	0.5%	1.2%	0.7%	
Race					0.696
White	77.9%	78.0%	76.9%	78.7%	
Black or African American	11.8%	10.6%	12.9%	12.0%	
American Indian or Alaska Native	2.5%	2.2%	3.2%	2.2%	
Asian	4.5%	5.8%	4.6%	3.1%	
Native Hawaiian or Pacific Islander	0.3%	0.2%	0.2%	0.5%	
Other	3.0%	3.1%	2.2%	3.6%	
Ethnicity					0.701
Non-Hispanic	89.0%	88.4%	90.0%	88.5%	
Hispanic	11.0%	11.6%	10.0%	11.5%	
Education					0.181
High school graduate or less	4.5%	3.6%	5.6%	4.3%	

Some college	*46.4%*	*49.3%*	*48.1%*	*41.9%*	
College graduate	*33.7%*	*32.6%*	*30.3%*	*38.0%*	
Graduate degree	*15.4%*	*14.5%*	*16.0%*	*15.8%*	
Political Affiliation					*0.013*
Democrat	*40.3%*	*42.9%*	*42.0%*	*35.9%*	
Republican	*34.1%*	*29.0%*	*37.1%*	*36.2%*	
Prefer not to say	*25.6%*	*28.0%*	*20.9%*	*27.9%*	
Household Income, annual					*0.669*
$0–$24,999	*27.6%*	*27.1%*	*28.9%*	*26.8%*	
$25,000–$49,999	*25.1%*	*26.2%*	*26.5%*	*22.7%*	
$50,000–$74,999	*16.6%*	*17.2%*	*14.6%*	*17.9%*	
$75,000+	*30.7%*	*29.5%*	*30.1%*	*32.5%*	

Race was measured via a self-selection question "What is your race? (check all that apply)"

**P-Value generated using Chi-Square test.*

Supplemental Table 2. Primary Outcome Measures and indicators.

Outcome	Indicator item wording [response scale]
Health Concern	*How much do these messages make you concerned about the health effects of eating red meat?*
	Not at all
	Very little
	Somewhat
	Quite a bit
	A great deal
Environmental Concern	*How much do these messages make you concerned about the environmental effects of eating red meat?*
	Not at all
	Very little
	Somewhat
	Quite a bit
	A great deal
Discouragement	*How much do these messages discourage you from wanting to eat red meat?*
	Not at all
	Very little
	Somewhat
	Quite a bit
	A great deal
Unpleasantness	*How much do these messages make eating red meat seem unpleasant to you?*
	Not at all
	Very little
	Somewhat
	Quite a bit
	A great deal

Supplemental Table 3. Secondary Outcome Measures and indicators.

Outcome	Indicator item wording [response scale]
Attention	How much do these messages grab your attention?
	Not at all
	Very little
	Somewhat
	Quite a bit
	A great deal
Negative Affect	How much do these messages make you feel scared?
	Not at all
	Very little
	Somewhat
	Quite a bit
	A great deal
Cognitive Elaboration	How much do these messages make you think about the environmental/health harms caused by eating meat?
	Not at all
	Very little
	Somewhat
	Quite a bit
	A great deal
Social Interactions	How likely are you to talk about this message with others in the next week?
	Not at all likely
	A little likely
	Somewhat likely
	Very likely
	Extremely likely
Intention to reduce meat consumption	Do you intend to reduce your red meat consumption in the next 30 days?
	Not at all likely
	A little likely
	Somewhat likely
	Very likely

		Extremely likely
Frequency of red meat consumption	*In the past 30 days, how often did you eat red meat?*	
		1 time per week
		2-3 times per week
		4-6 times per week
		1 time per day
		2 times per day
		3 or more times per day
